# Building the European Social Innovation Database with Natural Language Processing and Machine Learning

**DOI:** 10.1038/s41597-022-01818-0

**Published:** 2022-11-12

**Authors:** Abdullah Gök, Roseline Antai, Nikola Milošević, Wesam Al-Nabki

**Affiliations:** 1 Strathclyde Business School, University of Strathclyde, 199 Cathedral Street, Glasgow, G4 0QU United Kingdom; 2 Bayer AG, Müllerstraße 178, 13353 Berlin, Germany; 3 School of Computer Science, University of Manchester, Kilburn Building, Oxford Road, M13 9PL Manchester, United Kingdom; 4 Department of Electrical, Systems and Automation, Universidad de León, León, Spain

**Keywords:** Society, Business

## Abstract

Social innovation is widely defined as technological and non-technological new products, services or models that simultaneously meet social needs and create new social relationships or collaborations. Despite a significant interest in the concept, the lack of reliable and comprehensive data is a barrier for social science research. We created the European Social Innovation Database (ESID) to address this gap. ESID is based on the idea of large-scale collection of unstructured web site text to classify and characterise social innovation projects from around the world. We use advanced machine learning techniques to extract features such as social innovation dimensions, project locations, summaries, and topics, among others. Our models perform as high as 0.90 F1. ESID currently includes 11,468 projects from 159 countries. ESID data is available freely and also presented in a web-based app. Our future workplan includes expansion (i.e., increasing the number of projects), extension (i.e., adding new variables) and dynamic retrieval (i.e., retrieving and extracting information in regular intervals).

## Abstract (summary)

**Table:** 

Measurement(s)	social innovation projects
Technology Type(s)	natural language processing objective
Factor Type(s)	Project Scores (four variables) • Project Location • Project Topic • Project Summary • Project URL • Project Source
Sample Characteristic - Organism	N/A
Sample Characteristic - Environment	social innovation system
Sample Characteristic - Location	global

## Background & Summary

While it has many definitions, the European Union defines social innovation as the technological and non-technological new products, services or models “that simultaneously meet social needs and create new social relationships or collaborations”^1^. Some recent examples from the European Commission’s European Social Innovation Competition’s 2021 winners^2^ include SkillLab^3^, a mobile app that enable people to identify and present their skills, Snowball Effect^4^, a training programme that enables social entrepreneurs to replicate already successful social businesses, Zeki^5^, an online service that advices young people for support services, Happaning^6^, an immersive video service for events, and Mycotex^7^, a 3D manufacturing technology for sustainable and vegan textiles from mushroom roots. Historically, cooperative, hospice and fair-trade movements, public libraries, and open universities are well known examples of social innovation. Although social innovations can be technological, most of them do not utilise physical technologies but try to address unresolved social needs with novel social interactions. While the concept of social innovation has been in circulation since the 19^th^ century^8^, there is a growing body of literature that has been gathering attention from practitioners, policymakers, and social science researchers^9–11^ with over 100 new academic articles published each year. Social innovation is one of the emerging topics in innovation studies field, encompassing business and management, sociology, economics, and other social science disciplines.

One major issue that has dominated the field for many years is the availability of reliable and sustainable data. Unlike conventional innovation activity, which has established definitions, metrics and data sources^12,13^ that enable robust quantitative social science research, there has been a lack of a comprehensive data to study social innovation^14^. As a result, few published studies have provided quantitative evidence focusing specifically relating to social innovation^9,15^.

Since the early 2000s, there have been several attempts to create databases of social innovations, mainly funded by the European Union (for instance Digital Social Innovation Database^16^, Atlas of Social Innovation^17^ and Social Innovation in Marginalised Rural Areas Database^18^). However, most of these databases have suffered from inadequate sample sizes (ranging from 50 to 1,000 projects), often thematically focused (e.g., on a certain social issue like aging or on a specific technology such as digital social innovation) and an overemphasis on a particular definition of social innovation. One of the factors that limit the sustainability and quality of these databases is that these attempts were all based on human input through researchers conducting case studies of social innovation projects or social innovation projects registering themselves.

This gap motivated us to create the European Social Innovation Database (ESID), a global and comprehensive source of information on social innovation projects. Rather than being exclusively human inputted, ESID collects and processes its data through semi-automatic machine learning and natural language processing techniques, capitalising on the fact that social innovation projects, by nature, reveal considerable information about themselves on the publicly available web (e.g., websites or social media feeds). ESID contains over ten-times more projects compared to its predecessors, it has a flexible and innovative conceptual structure that encompasses the myriad of definitions of social innovation, and it is substantially more sustainable due to relatively limited human input. As ESID relies on human input less, it is less labour intensive and thus it is less prone to definition bias, especially considering that the concept of social innovation has many different definitions.

ESID is intended to serve to two audiences. First, social scientists studying social innovation that historically relied on small scale databases or qualitative methods, which was seen as a research barrier for the exponentially increasing research on social innovation^9–11^. ESID provides a comprehensive and valuable alternative by providing robust, transparent, and sustainable quantitative data. Second, ESID provides an opportunity to explore social innovations in various contexts such as type of social innovation, geography, topic to practitioners including policymakers, support organisations and social innovators themselves.

## Methods

**Fig. 1:**
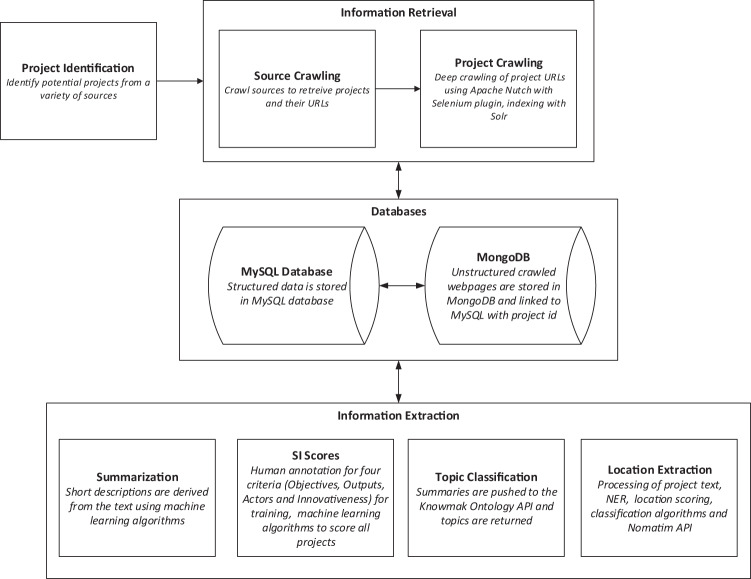
An overview of ESID Methodology.

We identified potential social innovation projects from a variety of sources. We, then, retrieved the textual data (e.g., websites) of these projects in an information retrieval stage. By using the textual data, we extracted and derived useful information such as topics, locations, summaries, and scores of social innovation projects. Unstructured textual data is stored in a MongoDB, while structured data is stored in a MySQL database. Each of these steps are detailed below (Fig. 1).

### Project identification

The first step in constructing our data was the identification of potential social innovation projects. We commenced by searching a range of relevant data sources such as the existing online human-inputted social innovation databases and repositories, which formed as a seed to train our machine learning models. We then initiated an additional open search phase where we gradually moved to indirect sources, for instance the European Union Social Innovation Competition^19^, a prize given to social innovation projects; Stanford Social Innovation Review^20^, a practice journal which includes articles and case studies mentioning social innovation projects; and Ashoka^21^, a social innovation community which contains a registry of its members. As the next step, we are planning to move to even more indirect sources such as large crowdfunding platforms not particularly specific to social innovation projects and at the end of the spectrum to the open web search.

### Information retrieval

We commenced our initial engagement with the data by scraping the sources containing potential projects. The overall objective here was to identify potential projects by utilising custom scripts based on Scrapy framework. This phase encompassed recording the potential projects identified to a MySQL database with the available information contained in these sources such as project title, URLs, short descriptions, countries, and cities where these projects are based, and other information where available.

For each potential project identified, we fed project URLs to an Apache Nutch v1.18 based system for deep crawling. The crawled unstructured website text indexed in Apache Solr 8.8.1. were then transferred to a collection in MongoDB, including metadata, (e.g., page URL, title, timestamp, etc.). The collection in MongoDB is linked to the MySQL database with a common project ID.

There were certain challenges associated with this; crawling is not always a straightforward process, as some of the project websites are temporarily or permanently unavailable, not amenable to crawling due to extensive use of JavaScript or crawl-protection technologies or containing too little or too large amount of text. This requires extensive human supervision and data cleaning. To address some of these crawling problems, we also implemented an additional layer of Apache Nutch system that uses a Selenium plug-in, which allows us to combine the robustness and scalability of Apache Nutch with the Java Script handling capabilities of Selenium.

### Information extraction

#### Social innovation scores

In the next stage, we derived several features/variables from the unstructured text of the social innovation project websites. The foremost feature is a set of scores measuring the “social innovativeness” of a project. The definition of social innovation varies in the literature and a precise definition of the concept has proved elusive. As a result, the meaning of this term has been a matter on ongoing discussion in the literature, where a substantial part of the research on the topic is exclusively devoted to the precise definition of the concept, while almost all empirical studies spend considerable amount of effort to justify the definition they employ.

A review of several studies that surveyed different meanings of the modern concept reveals four main elements that define social innovation^9,22–29^. While nuances between definitions vary, these broad criteria generally apply:

i. **Objectives:** Social innovations satisfy societal needs - including the needs of particular social groups (or aim at social value creation) - that are usually not met by conventional innovative activity (c.f. “economic innovation”), either as a goal or end-product. As a result, social innovation does not produce conventional innovation outputs such as patents and publications.
ii. **Actors and actor interactions:** Innovations that are created by actors who usually are not involved in “economic innovation,” including informal actors, are also defined as social innovation. Some authors stress that innovations must involve predominantly new types of social interactions that achieve common goals and/or innovations that rely on trust rather than mutual-benefit relationships. Similarly, some authors consider innovations that involve different action and diffusion processes but ultimately result in social progress as social innovation.
iii. **Outputs/Outcomes:** Early definitions of social innovation strongly relate it with the production of social technologies (c.f. innovation employing only “physical technologies”) or “intangible innovation.” This is complemented by some definitions, which indicate that social innovation changes the attitudes, behaviours and perceptions of the actors involved. Some other definitions stress the public good that social innovation creates. Social innovation is often associated with long-term institutional/cultural change.
iv. **Innovativeness:** Innovativeness is generally used to differentiate social innovation from social entrepreneurship. This covers not only technological but also non-technological innovation.

Rather than adapting a particular definition, ESID employs a flexible conceptual structure in which we disentangle the concept of social innovation on the basis of the above four definition components^30^. ESID assigns each project a score for each of the i) objectives, ii) actor and actor interactions, iii) outputs/outcomes, and iv) innovativeness criterion, thus each project has four social innovation scores. The scoring system we developed enables users to filter the social innovation projects based on their preferred definition, while it is also critical to verify the quality of the projects identified, as some of the projects included from some of the sources score very low for all the criteria.

For each of these criteria, we created supervised machine learning models which are trained to predict the scores for each criterion. A manual annotation of about 20% of projects is used to train/evaluate our models. New annotations were added whenever we included projects from a new data source to generate a training set that is representative of our dataset, but we always used the same annotation guidelines and moderator to ensure the consistency. We experimented with various human annotation approaches, including annotating sentences indicating each of the above mentioned four social innovation criteria as well as assigning scores to each of them in several annotation workshops. We observed that project level scores were more reliable, efficient, and robust than sentence annotation for our models. For scoring, we also experimented with various scoring schemas (5-bin, 3-bin, 2-bin) while we settled a 3-bin scoring (0: no indication of the criteria, 1: partial indication of the criteria, 2: full indication of the criteria) as it ensured the optimum inter-annotator agreement and model performance. Annotation criteria and guidelines are presented in Tables 1, 2.

**Table 1:** Annotation Criteria.

Element of Definition	Criteria
1. Objectives	Project primarily or exclusively satisfies (often unmet) societal needs, including the needs of particular social groups; or aims at social value creation.
	Often no price is involved for the beneficiary, or the innovation is provided to the beneficiary at low cost without any profit motive. However, there are examples where a fee is involved.
2. Actors and Actor Interactions	• Satisfy one or both of the following sub-criteria: i. Diversity of Actors: Project involves actors who would not normally be involved in innovation as an economic activity, including formal (e.g., NGOs, public sector organisations etc.) and informal organisations (e.g., grassroots movements, citizen groups, etc.). This involvement might range from full partnership (i.e., project is conducted jointly), to consultation (i.e., there is representation from different actors). ii. Social Actor Interactions: Project creates collaborations between “social actors” (i.e., actors that are not conventional innovation creators, but engage in social innovation such as charities, social enterprises, public sector organisations), small and large businesses and public sector in different combinations. These collaborations usually involve (predominantly new types of) social interactions towards achieving common goals such as user/community participation. Often, projects aim at significantly different action and diffusion processes that will result in social progress. Often social innovation projects rely on trust relationships rather than solely mutual benefit.
3. Outputs and Outcomes	Project primarily or exclusively creates socially oriented outputs/outcomes. Often these outputs/outcomes go beyond those created by conventional innovative activity (e.g., products, services, new technologies, patents, and publications), while conventional outputs/outcomes might also be present. These outputs/outcomes are often intangible, and they might include the following but not limited to: • change in the attitudes, behaviours and perceptions of the actors involved and/or beneficiaries • social technologies (i.e., new configurations of social practices, including new routines, ways of doing things, laws, rules or norms) • long-term institutional/cultural change
4. Innovativeness	The Project should include the implementation of a new or significantly improved product (good or service), or process, a new marketing method, or a new organisational method.
	The project needs to include some form of innovative activities (i.e., scientific, technological, organisational, financial, and commercial steps intending to lead to the implementation of the innovation in question). Innovation can be technological (involving the use of or creating technologies) as well as non-technological.
	The innovation should be at least “new” to the beneficiaries it targets (it does not have to be new to the world).

**Table 2:** Annotation Guidelines.

Element	Guidelines
Scores	• 2: fully satisfies the meaning of the criteria • 1: partially satisfies • 0: no indication at all
Claims versus evidence	• We will assess what the project claims and will not seek any evidence of achievement. Some of the things might not happen yet (still being planned), but it is enough for us if they are mentioned as plans.
Guidelines for actors and actor interactions criteria	• If one of the sub-criteria is fully satisfied, please grade as 2. • If both sub-criteria are partially satisfied (normally grade 1) please grade as 2 (since both of them are satisfied).
Guidelines for outputs/outcomes criteria	• If the project claims they are conducting an innovation by using the term, but they do not substantiate the nature of innovations described above, give score 1.
Guidelines for innovativeness criteria	• The project does not have to use the term “innovation” explicitly but if it satisfies the above criteria without using the term at all, it still might be marked as 2.

Utilising the human annotation, we experimented with a variety of different types of supervised models and specifications, while we obtained the best results (i.e. F1 score around 0.90) with a Bidirectional Encoder Representations from Transformers (BERT) language representation model^31^ (see Table 3) using a 3-bin classification (0,1,2). Using BERT requires fine-tuning on a small dataset of the designated classes. To this end, we used 5,162 projects, 80% of the dataset for training and 20% for testing. Before the classification, we pre-processed the text by applying the following: (i) removing HTML tags if exists and special characters like #, =, and &, (ii) eliminating long sequence of characters, i.e., more than 20 characters, and (iii) dropping duplicated sentences but with maintaining the order of the text. To train BERT classifier we fine-tuned BERT base cased version using the SimpleTransformers framework^32^.

**Table 3:** Performance of Social Innovation Criteria Models.

Type of Model	Objectives			Actors and Actor Interactions			Outputs/Outcomes			Innovativeness		
	Precision	Recall	F1	Precision	Recall	F1	Precision	Recall	F1	Precision	Recall	F1
TFIDF LR	0.78	0.73	0.75	0.86	0.77	0.80	0.83	0.77	0.79	0.81	0.75	0.77
TFIDF NB	0.86	0.68	0.67	0.90	0.72	0.76	0.89	0.71	0.74	0.90	0.72	0.74
TFIDF SVM	0.88	0.74	0.75	0.89	0.75	0.79	0.88	0.72	0.76	0.86	0.74	0.76
BERT	0.90	0.90	0.90	0.91	0.91	0.91	0.90	0.90	0.90	0.88	0.88	0.88

The best results were yielded by fine-tuning BERT followed by the LR classifier (Table 3). BERT model has state-of-the-art results in several Natural Language Processing tasks^33,34^. BERT is a context-based transformer language model that generates word representations for each word based on distributional semantics hypothesis from linguistics^35^. The hypothesis says that words that are used and occur in the same contexts tend to communicate similar meanings. The word representations in BERT are based on an unsupervised language model over large amount of text, where certain words are masked, and the task of the model is to predict masked words based on the surrounding context. Contextuality, attention mechanism^36^ that gives importance to certain portion of the context and the depth of the model in BERT contribute to its superiority compared to previous approaches.

#### Summarisation

**Fig. 2:**
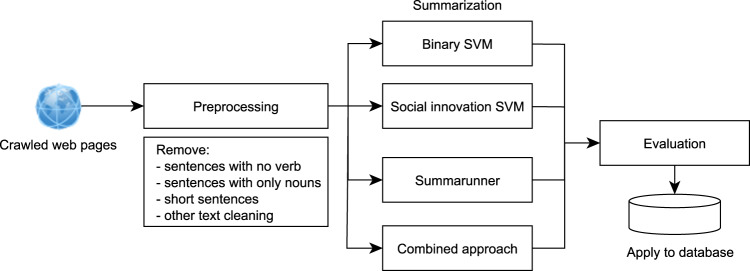
Summarisation Methodology.

Availability of the short descriptions of projects is important for two reasons. First, short descriptions provide a quick snapshot to the users and offer them the opportunity of extracting further features through topic modelling. Second, the BERT model we employed to score the four social innovation criteria requires a relatively short text length (>512 tokens). To obtain short descriptions which are representative of the text, we experimented with three different models, and we used a combination of these (see Figure Fig. 2).

- Support Vector Machine (SVM) is a machine learning algorithm that works on high dimensional datasets and works by finding the best hyperplane that separates the datasets into the most differentiative classes. The central idea in SVM is the choice of the hyperplane which sits “right in the middle” of two classes, or more mathematically, it chooses the hyperplane that maximizes the minimum distance between the hyperplane and all the examples^37^. In the Binary SVM summarisation implementation, we worked on the assumption that the summarisation task can be modelled as a classification task, where constituent sentences in the text were classified into either being part of the summary or not. It was hypothesized that words in a sentence could indicate whether it described the project (e.g., “project aims to…”, “the goal of the project is to…”, etc.) or not. A training set was created using descriptions obtained from original project sources and the unstructured crawled texts of each website. Cosine similarities were computed for between the sentences from the description and those from the crawled text and if the similarity score was above 0.8, the sentence was labelled as part of the summary, and vice versa. These sentences were then used to train the SVM algorithm to predict which sentences should become part of the summaries^37^.
- For the Social Innovation criteria classifier, an annotated dataset was utilised. The dataset consisted of sentences which were marked as explaining why a project satisfies any of the social innovation criterion. These were used as positive training instances for the SVM classifier.
- Summarunner is an extractive summarisation method, developed by IBM Watson, and it utilises recurrent neural networks (GRU). This method visits sentences sequentially and classifies each sentence by whether it should be in the summary. It uses a 100-dimensional word2vec.
- Our combined approach comprised of the SVM-based method and Summarunner. We observed that the binary SVM model produced quite long summaries, and this was unsuitable for our goal of generating summaries. As such, we utilised that approach for the initial cleaning of the text, and once this was completed, we utilised Summarunner in shortening the text to generate the final summary^38^. This combined approach gave the best results for our summarisation task.

**Fig. 3:**
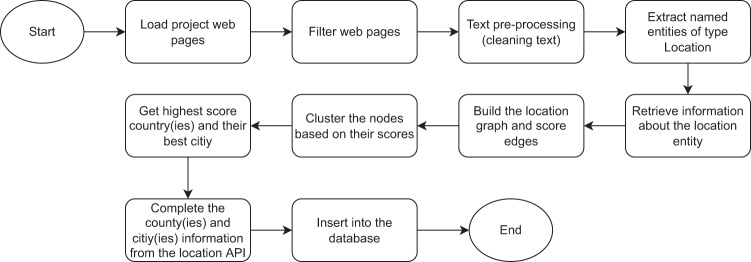
Flowchart of the Location detection algorithm.

Location of the projects is an important information to study the territorial and policy dynamics. Some of the sources already include information on the location of the projects in a higher granularity (e.g., as country) while as we progress into identifying potential projects from more indirect sources, location information becomes unavailable from the sources we identify projects. This motivated us to extract locations from the corpus of projects. In this process, we encountered three problems. First, for some projects location is not mentioned in the text at all. Second, in some projects there are numerous locations mentioned but which one of these locations are the location of the project is not clear. Third, when a location is mentioned, sometimes it is not complete (i.e., only a city mentioned with no country information, but there exist various cities with the same name in various countries). To overcome these problems, we developed an algorithm that uses Named Entity Recognition (NER) to identify locations and Graph Theory to analyse the inter-connectivity between these location entities (Fig. 3), with the following key steps:

- **Text pre-processing:** as some projects have vast amount of text, we cleaned duplicates and selected the most possible pages that might contain the “correct” location of the project such as the home page, about us or contact us pages (or their string variations in the page title or URL).
- **Location Entity Extraction**: after having the text pre-processed, we use Named Entity Recognition (NER) model to identify entities of the type “Location”. We used a state-of-the-art NER model, called “ner-english-ontonotes-large”^39^. The model is based on Transformers architecture and trained on the Ontonotes dataset. After extracting all the entities, we aggregate them based on their frequency for further processing.
- **Location Scoring**: after extracting all the location named entities, the next step is to filter them by removing irrelevant ones, i.e., those which do not represent the project location. For each location entity, we call an online API, called Nominatim^40^ that depends on OpenStreetMap (OSM) data to extract meta-data about the called location entity. Nomanatim is widely used in the study of innovation in geographical context. The API returns a JSON file per request with a list of candidates. Each candidate holds the following keys: corresponding country name, country name in Alpha-3 code encoding, importance score based on OSM ranking algorithm, and location type, whether a city, country, village, suburb, or region. We split the locations into “County” and “City” based on the location type. At this stage, each location has an importance score, frequency score, and type, but we still do not know the correct project location. We used Graph Theory to represent the extracted locations along with their meta-data as a directed weighted graph that consists of nodes and directed edges. First, we create the nodes of type “Country”. Next, for each location of type “City”, we find its potential corresponding countries, thanks to the Nominatim API response, and we created a directed link from the city to its potential counties. An edge between a given city and its country is weighted following the next equation:
  country_city_edge_weight = alpha * city_importance * city_frequency + beta * country_frequency,
  Alpha and beta are adjustable hyperparameters to control the impact of the city and country on the edge’s weight. We set them to 0.7 and 0.3, respectively, as city provides more specific and useful information. Next, each country receives a score equal to the sum of its incoming edges’ weights.
- **Location aggregation:** a project could take place in one or more countries, and the algorithm should be able to decide these locations. To this end, we use the MeanShift^41^ algorithm to cluster countries based on their scores into groups. The group with the highest score is the most probable location(s).
- **Location Information Retrieval**: once we extract the city and country, we retrieve their information using the Location API, Nominatim, which we already cached during the scoring step. We extract the following: city name, city type, country name, country type, country ISO alpha-3 name, longitude and latitude of both city and country, and city and country wikidata IDs.
- **Information storage**: once the information is extracted, we insert them into a table in our MySQL database.

The performance of our location detection algorithm is presented in Table 4.

**Table 4:** Performance of Location Detection Algorithm.

Item	Value
Number of projects with city mentioned in text	1511
Number of projects with city mentioned in text and has ground truth	958
Accuracy - city only	63%
Number of projects with country mentioned in text	1558
Number of projects with country mentioned in text and has ground truth	1402
Accuracy - country only	90%
Number of projects with city or country mentioned in text	1973
Number of projects with city or country mentioned in text and has ground truth	1749
Accuracy - city or country	89%

#### Topics

We tagged projects with an ontology of topics developed as part of the KNOWMAK project^42^. The ontology has two main classes of Key Enabling Technologies (KET) topics and Societal Grand Challenges (SGCs) based on the EU H2020 priorities. The topics are hierarchical and there are two sub-levels for each of these main classes. We used the KNOWMAK Ontology API to classify topics by pushing the summaries obtained in the previous step. The API then returned to us a number of topics associated with each project, as well as assigned scores. We set a threshold for each of the scores and assigned the topics with scores above that threshold as the topics for the project.

### Future development

The present study lays the groundwork for future tasks which is underway to improve ESID in the following three areas:

#### Expansion

In the next stage, we are initiating additional open search phases by exploiting more indirect sources, such as crowdfunding platforms. Whilst this will bring additional information retrieval challenges as these platforms usually do not allow programmatic access, it will expand the database by providing a comprehensive list of social innovation projects.

#### Extension

We are also planning to add two main features. First, we will work with the actors (e.g., organisations related to the projects) mentioned in the project websites. These actors will be classified by their organisational type (university, public sector, third sector, etc.) and their relationship to the projects (project owner, funder, partner, etc.). Second, we will extract the objectives (i.e., the social problems these projects aim to address, e.g., climate change), activities (activities do they conduct to achieve their objectives, e.g. reducing food waste) and accomplishments (tangible accomplishments achieved, e.g. reducing food waste by a certain amount). We will extract the sentences related to each of these three features and subsequently we will run a topic model to classify these features into categories.

#### *Dynamic* retrieval

Currently ESID crawls the project websites and extracts variables related to them only once. We are planning to re-crawl the project websites at regular intervals to identify the changes in these variables, which would then provide us with a time-series data.

## Data Records

**Fig. 4:**
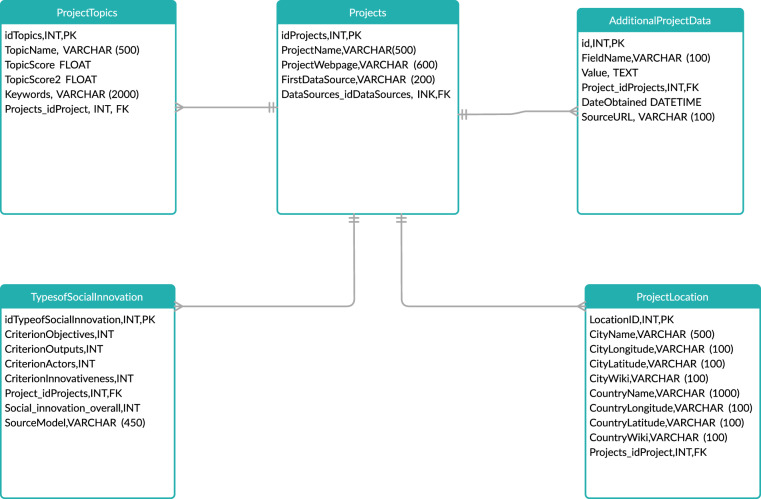
Simplified Schema.

The main entity in ESID is social innovation projects. For each project, ESID currently includes the following main variables (a simplified version of the Database schema is presented in Fig. 4):

- Social Innovation Scores: This consists of a score for each of the social innovation criteria explained above. Thus, each project has a score for objectives, actors and actor interactions, outputs, innovativeness. Each score ranges between 0 (no indication of the criteria), and 2 (fully satisfies the meaning of the criteria).
- Location: We identify location as city and country. Subsequently we derive the central coordinates, from which we populate other location information such as Wikidata ID.
- Summary: a short summary of the project, either used from the original data source or summarised by our summarisation model.
- Topics: a set of topics associated with each project. Topics are grouped as Key Enabling Technologies (KETs) and Societal Grand Challenges as defined by the EU H2020 priorities.

The current version of ESID is available at a figshare repository under a CC-BY licence^43^. It includes 4 csv files as well as a 5^th^ helper file that indicates the topic mapping:

- Basic project info: Project_id; Project Title; Project URL; Project Source
- Projects and Scores: Project_id; Scores for the criteria of Objectives, Actor and Actor Interactions, Innovativeness, Outputs; Source of Scores (human annotation or model prediction)
- Projects and Country: Project_id; a number of location-based columns
- Topics: Project_id; Topic Name and scores

ESID currently includes 11,468 projects. Some of these projects are *negatives*, i.e., projects that score 0 for all four social innovation criteria and thus cannot be considered as social innovation by any definition. However, they play an important role in balancing our machine learning models by providing negative examples. Similarly, some projects have missing information (i.e., they have the country information but not the city). There are 6,692 projects with all the variables available.

**Fig. 5:**
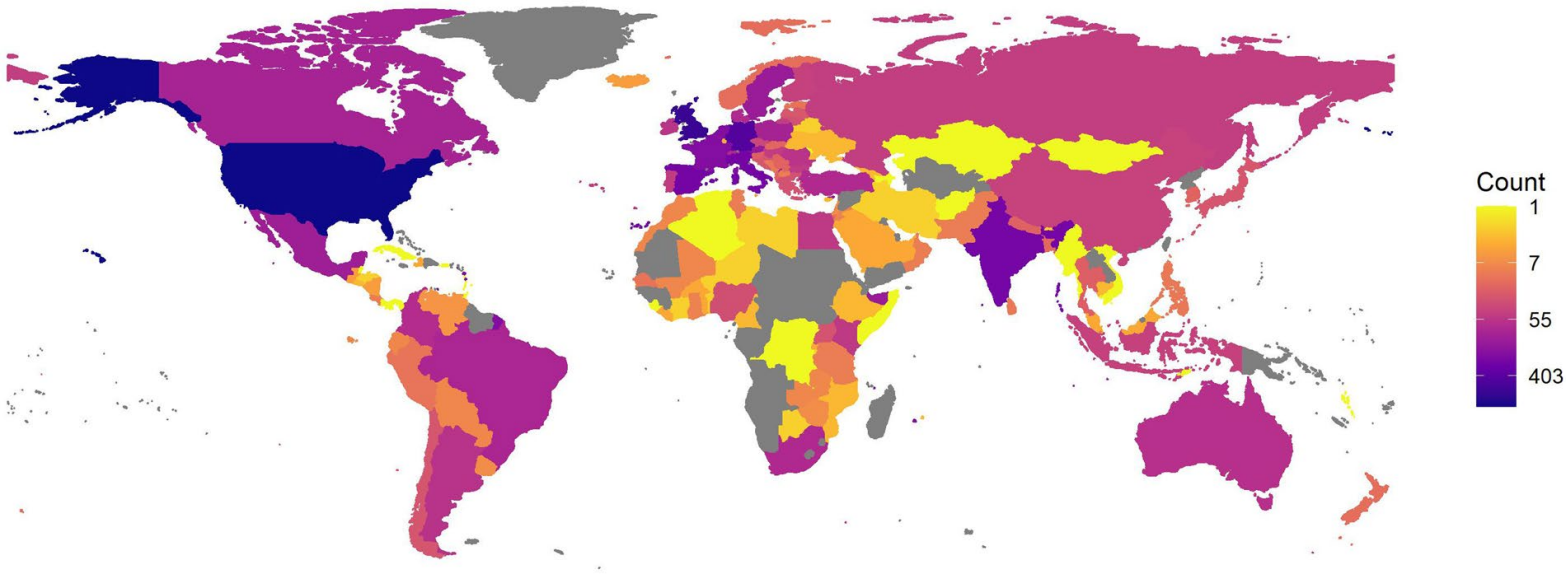
Geographical Coverage.

Even though the title of the database implies a European orientation, ESID includes data from 159 countries. The projects located in the EU countries consists of about one third of the database, while projects in non-EU Europe (including the UK) and North America are the next most represented geographies (both are about 10% of the data) (see Fig. 5).

**Fig. 6:**
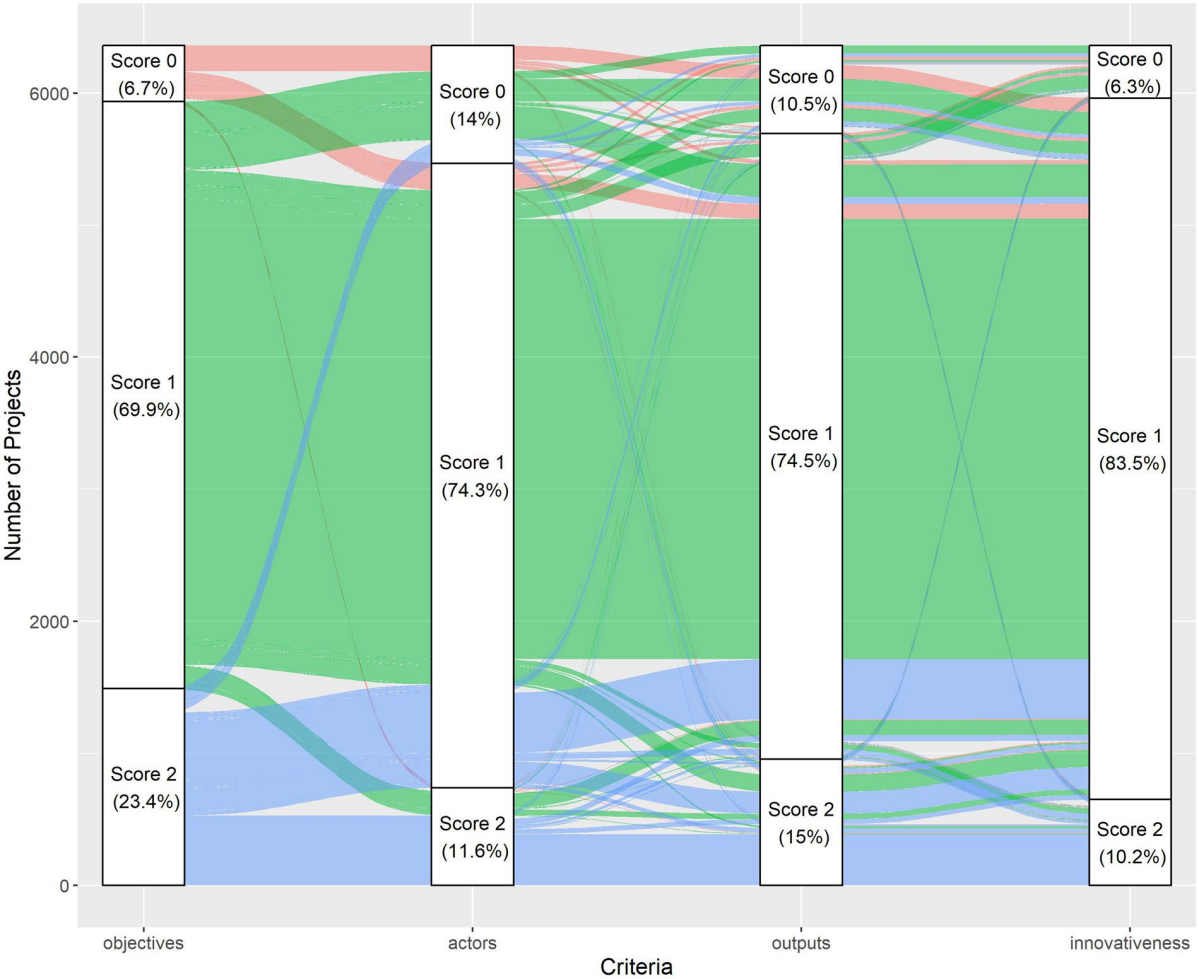
Score Distribution.

Note: Only the projects that have a score greater than 0 in at least one criterion are included (i.e., “negative” projects excluded).

Arguably the most novel aspect of ESID is that it does not have a preferred definition of a concept whose definition is highly debated. Rather ESID disentangles the social innovation definition into four components and scores projects on the basis of them. The distribution of scores indicates that the four criteria is distinct (>0.40 pairwise correlation) and there is a reasonable spread of scores (see Fig. 6). This novel approach enables users to filter the data according to their own definition. This also enables us to include projects that would not be considered as social innovation by exclusively adopting certain definitions. For instance, while the official EU definition stresses the criteria objectives and actors, about 20% of the projects scores 0 for these criteria but scores highly for outputs and innovativeness.

## Technical Validation

As ESID relies on the initial data sources as seed, especially in the first stage, there were some missing information. Similarly, while our models tried to balance between precision and recall as much as possible, there was inevitably a recall loss due to our classification scheme. To address these and increase our recall and precision, we employed the following manual mechanisms:

- Human Annotation: ESID classification models, particularly for the four social innovation classifiers, depend on extensive manual annotation to train and evaluate the models. The annotation process was conducted on an iterative basis. First, we started with sentence level extensive annotation and utilised data from six simultaneous annotators through annotation workshops. Following this, we moved to a project level annotation in the form of inputting scores. We experimented with various scoring schemas. We conducted an extensive analysis of these experiments and we concluded the optimum results were obtained with a system in which we use a three-point scoring system (0,1,2) at the project level. We also concluded that utilising a single annotator moderated by another independent annotator results in the optimal inter-annotator agreement and model performance. As we gradually developed our annotation process, we also developed extensive annotation guidelines (Table 1). Full details are available at the ESID Manual^44^.
- Manual checks: We also manually checked a substantial number of projects with an aim to increase the precision of ESID models. By checking the information provided on the projects, for instance, we could see if a website was taken over by another organisation, or if the summaries reflect the projects, etc.
- Augmenting or Adding data sources based on underrepresented social innovation topics: We analysed the database in terms of the major groupings of project themes (for instance, poverty reduction, refugee integration, environmental protection). For currently underrepresented groupings we investigated new sources, or we manually identified projects through web search.
- Quality control by stakeholders: We held a virtual workshop with stakeholders including our partners at the KNOWMAK project where each stakeholder investigated a subset of the ESID. Stakeholders were asked to review projects in the database and to make suggestions on the addition of missing projects or exclusion of irrelevant projects. This was then reviewed by the team and adjusted in ESID.
- Hackathon: We invited scholars and PhD students working on social innovation to a Hackathon Day. They were then encouraged to find errors and omissions.

These steps enabled us to create a high-quality seed set, which subsequently led to higher performance machine learning models.

For the quality check, we developed a web interface through which these reports were made. All the submissions to this interface were then subsequently reviewed manually before being updated in the database.

The text contained in the websites are mainly in English language, but ESID also covers non-English speaking countries and thus a portion of the project websites are in non-English languages. We used a language detection script to identify the language of each page. Currently, we are utilizing Apache Nutch’s language detection and have also added a language field in the Apache Solr index, where the abbreviations of each page’s language are shown. This is data is subsequently transferred to MongoDB. If there are English language pages within a website of a project (i.e., an English language section), in principle we use them. However, if there are no English language pages, we translate all the pages of a project website to English by using Google Translate, which performs reasonably well for our purposes, especially for the widely used languages.

## Usage Notes

ESID is a comprehensive and reliable data source for the study of social innovation, a rapidly growing practice that is garnering increased social science attention. Among the topics ESID is readily useful are the study of geography of social innovation, diffusion of social innovation, social innovation policy, emerging topics in social innovation. As ESID is a flexible and sustainable source, new variables can also be extracted in future from the project web text stored, addressing developing research topics.

ESID is also published as part of the EU-funded Research Infrastructure for Science and Innovation Policy Studies (RISIS) Project, where the most recent version is regularly updated. Users need to register with the RISIS Datasets Portal (https://rcf.risis2.eu/datasets) and put forward an access request outlining the use case and data needed. As part of the RISIS project, there are some funds available to support users travel expenses should they want to receive training and support in person. This needs to be indicated at the access request form.

ESID has also a publicly available app to browse and analyse the source data at https://bit.ly/ESIDapp.

## Data Availability

All the code is freely available in Github at https://github.com/EuropeanSocialInnovationDatabase/ESID_V2.
